# Contribution of Lysosome and Sigma Receptors to Neuroprotective Effects of Memantine Against Beta-Amyloid in the SH-SY5Y Cells

**DOI:** 10.34172/apb.2020.055

**Published:** 2020-05-11

**Authors:** Mojtaba Keshavarz, Majid Reza Farrokhi, Elahe Amirinezhad Fard, Mohammad Mehdipour

**Affiliations:** ^1^Shiraz Neuroscience Research Center, Shiraz University of Medical Sciences, Shiraz, Iran.; ^2^Department of Neuroscience, School of Advanced Medical Sciences and Technologies, Shiraz University of Medical Sciences, Shiraz, Iran.

**Keywords:** Amyloid beta-Peptides, Lysosomes, Memantine, Neuroprotection, Sigma receptors

## Abstract

***Purpose:*** Memantine is an approved drug for the treatment of Alzheimer’s disease (AD). Autophagy, lysosome dysfunction, and sigma receptors have possible roles in the pathophysiology of AD. Therefore, we aimed to investigate the contribution of sigma receptors and lysosome inhibition to the neuroprotective effects of memantine against amyloid-beta (Aβ)-induced neurotoxicity in SH-SY5Y cells.

***Methods:*** We determined the neuroprotective effects of memantine (2.5 µM), dizocilpine (MK801, as a selective N-methyl-D-aspartate (NMDA) receptor antagonist) (5 μM) against Aβ25– 35 (2 μg/μL)-induced neurotoxicity. We used chloroquine (10, 20, and 40 μM) as a lysosome inhibitor and BD-1063 (1, 10, and 30 μM) as a selective sigma receptor antagonist. The MTT assay was used to measure the neurotoxicity in the SH-SY5Y cells. Data were analyzed using the one-way ANOVA.

***Results:*** Memantine (2.5 µM), dizocilpine (5 µM), chloroquine (10 and 20 µM) and BD-1063 (1, 10 and 30 µM) decreased the neurotoxic effects of Aβ on the SH-SY5Y cells. However, chloroquine (40 µM) increased the neurotoxic effects of Aβ. Cell viability in the cells treated with memantine + Aβ + chloroquine (10, 20, and 40 μM) was significantly lower than the memantine + Aβ-treated group. Moreover, cell viability in the memantine + Aβ group was higher than the memantine + Aβ + BD-1063 (10 and 30 μM) groups.

***Conclusion:*** The lysosomal and sigma receptors may contribute to the neuroprotective mechanism of memantine and other NMDA receptor antagonists. Moreover, the restoration of lysosomes function and the modulation of sigma receptors are potential targets in the treatment of AD.

## Introduction

Neurodegeneration in several brain regions including neocortex and hippocampus may cause of the cognitive deficits in patients with Alzheimer’s disease (AD). Amyloid-beta (Aβ)-induced impairments in the activity of glutamate and autophagy systems may contribute to the neurodegeneration in the central nervous system (CNS).^1,2+^ Therefore, the restoration of homeostasis in the glutamate and autophagy systems may help block the Aβ-induced neurotoxicity.

Memantine is a non-competitive antagonist of the N-methyl-D-aspartate (NMDA) receptors of glutamate approved for the treatment of AD.^3^ Some human studies and animal models have shown its neuroprotective and cognitive-enhancing effects in the AD.^4,5^ Other systems alongside with the NMDA receptors may have a role in the therapeutic effects of memantine.^6^ By considering the possible roles of autophagy and sigma receptors in the pathophysiology of the AD,^7,8^ these systems may be involved in the mechanism of action of memantine.

Autophagy is a cellular homeostatic process that degrades noxious proteins and damaged organelles.^9^ This process is mainly dependent on lysosomes.^10^ Lysosomes are the specialized organelles contained hydrolase enzymes such as proteases and lipases.^11^ This organelle is closely related to the digestive activity of the autophagy process.^9^ Lysosome blockade is among various autophagy dysfunctions contributed to the neurodegeneration.^12^ Chloroquine is a lysosomotropic agent which is widely used for the autophagy inhibition in the experimental models.^13^ This agent increases the pH of lysosome and inhibits the function of lysosomal enzymes.^14^ The Aβ-induced over-activation of the NMDA receptors leads to the autophagy activation and neuronal apoptosis.^15^ However, the contribution of lysosome inhibition to the neuroprotective effects of NMDA antagonists like memantine is elusive.

Sigma receptors are widespread in the CNS and they are involved in different brain functions such as learning and memory, neuroprotection and immunomodulation.^16,17^ Various experimental models have documented the neuroprotective effects of the sigma receptors in brain injury and neurodegeneration.^18^ Sigma receptors regulate several neurotransmitter systems such as glutamatergic, noradrenalinergic, dopaminergic, and cholinergic systems.^19^ Although the NMDA receptors can be modulated by the sigma-1 receptors,^19,20^ the interaction between these two receptors has remained controversial.^21^ Some studies have shown that the activation of the sigma receptor produces neuroprotection by interfering with the intracellular machinery of NDMA receptors.^22^ By considering the role of the NMDA receptors in the neurotoxic effects of Aβ peptide^1^, the sigma receptors may have a possible role in the neuroprotective effects of NMDA antagonists like memantine. In this study, we aimed to explore the contribution of sigma receptors and lysosome inhibition to the neuroprotective effects of memantine against Aβ-induced neurotoxicity in the SH-SY5Y cells.

## Materials and Methods

### Materials

The human SH-SY5Y neuroblastoma cells were obtained from the Pasteur Institute (Iran). We purchased Dulbecco’s modified Eagle’s medium and Ham’s nutrient mixture F-12 (DMEM/F12), fetal bovine serum (FBS), and Penicillin-Streptomycin from Gibco^®^life technologies^™^(USA). Moreover, Aβ25-35, memantine, chloroquine, and BD-1063 (a selective sigma receptor antagonist) were obtained from the Sigma-Aldrich (USA).

### Neuronal cell culture 

We used SH-SY5Y neuronal cells to determine the neurotoxic and neuroprotective activities of the administered agents. We seeded the SH-SY5Y cells at the density of 1 x 10^5^ cells/well in the 96-well plates. The cell culture medium contained DMEM/F12 (1:1), fetal bovine serum (10 %), penicillin (100 U/mL), and streptomycin (100 µg/mL). The cells were kept in a standard condition of the humidified atmosphere of 95% air/5% CO2 and 37°C.

### Study design and treatments

We used the fibril form of Aβ to conduct the neurotoxicity test. Aβ25–35 (2 μg/μL) was incubated in the water bath for four days at 37°C to induce the aggregation process. Memantine (2.5 µM), dizocilpine (MK801, as a selective NMDA antagonist) (5 μM), chloroquine (10, 20, 40 µM), BD-1063 (1, 10, 30 μM), and Aβ (20 μM) were dissolved in the sterile water. We selected the concentrations of each agent according to the previous studies and a pilot study. The cells were treated with Aβ with or without memantine, dizocilpine, chloroquine, and BD1063 for 24h. The procedure was repeated for four samples.

### Cell viability assay

The 3-[4,5-dimethylthiazol-2-yl]-2,5-diphenyl tetrazolium bromide (MTT) was used to measure the neuronal viability. In brief, the MTT reagent (5 mg/mL) was poured into the wells containing the treated cells. After 4 hours, the media was removed and the precipitate dissolved in dimethyl sulfoxide (DMSO) (100 µL). The absorbance of each well was determined at 570 nm by a microplate reader (Synergy HT, Biotek^®^) as an index of cell viability.

### Statistical analysis

The results were analyzed using the one-way analysis of variance (ANOVA) test, followed by the LSD test as the post hoc test. *P* values <0.05 was considered statistically significant. We used the SPSS software (version 23) to analyze the data.

## Results and Discussion

### The effects of beta-amyloid, memantine, dizocilpine, chloroquine, and BD-1063 on neuronal survival

**Figure1:**
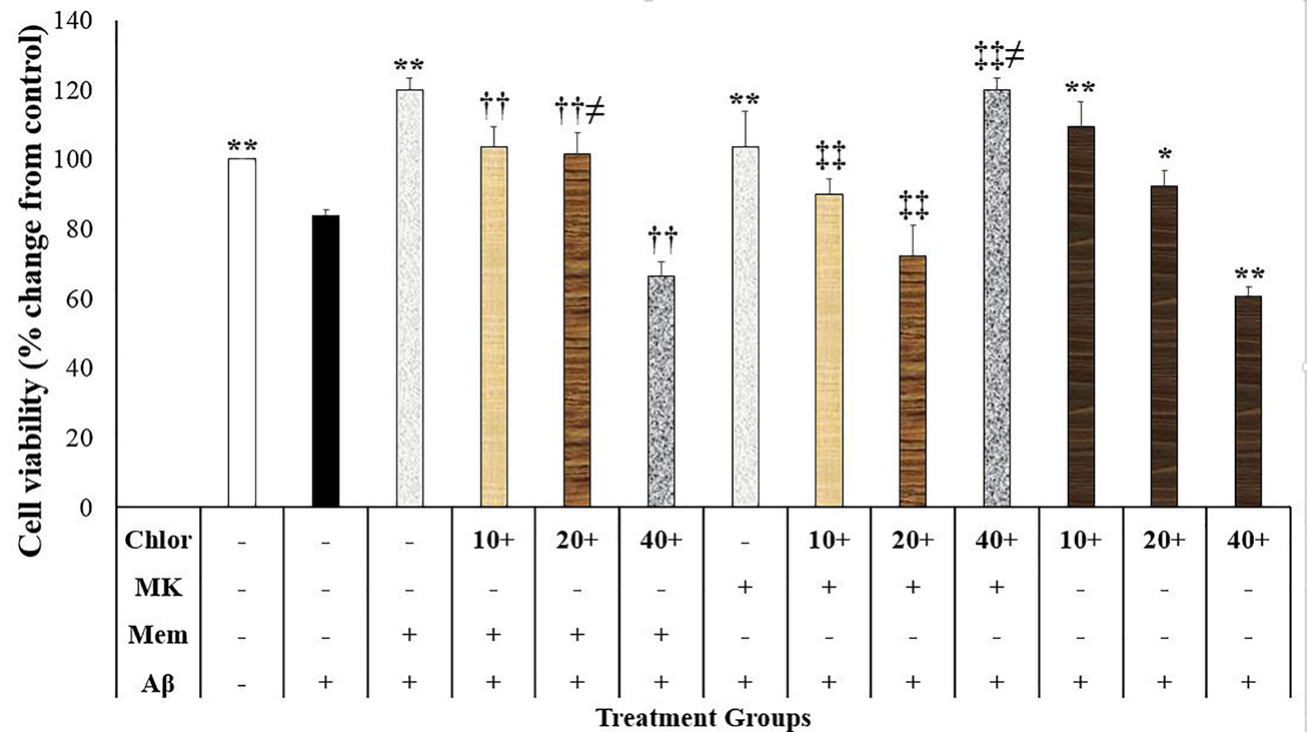
The effects of N-methyl-D-aspartate antagonists and chloroquine on the beta-amyloid (Aβ)-induced neurotoxicity in the SH-SY5Y cells (N=4). Data were analyzed using the one-way analysis of variance (ANOVA) followed by the LSD test. Cell viability was measured using the 3-[4,5-dimethylthiazol-2-yl]-2,5-diphenyl tetrazolium bromide (MTT) test. ** *P*  < 0.001 compared to the Aβ-treated group, †† *P*  < 0.001 compared to the Memantine + Aβ-treated group, ^‡‡^*P*  < 0.001 compared to the MK801+Aβ-treated group, and ^≠^*P*  < 0.05 compared to the Aβ + chlorloquine-treated group. Chlor: chloroquine, MK: dizocilpine, Mem: memantine, Aβ: beta-amyloid.

**Figure 2:**
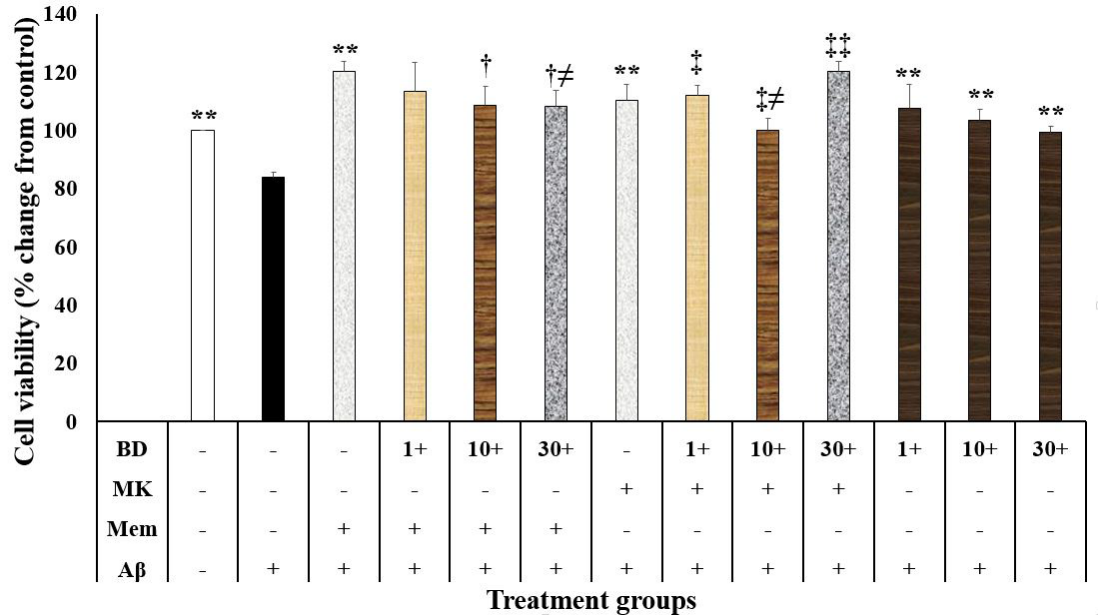
The effects of N-methyl-D-Aspartate antagonists and a sigma receptor antagonist on the beta amyloid (Aβ)-induced neurotoxicity in the SH-SY5Y cells (N=4). Data were analyzed using the one-way analysis of variance (ANOVA) followed by the LSD test. Cell viability was measured using the 3-[4,5-dimethylthiazol-2-yl]-2,5-diphenyl tetrazolium bromide (MTT) test. ** *P*  < 0.001 compared to the Ab-treated group, † *P*  < 0.005 compared to the Memantine + Aβ-treated group, ‡ and ‡‡ *P*  < 0.05 and *P*  < 0.001 compared to the MK801+Aβ-treated group, and ≠ *P*  < 0.05 compared to the Aβ + BD1063-treated group. BD: BD1063, MK: dizocilpine, Mem: memantine, Aβ: beta-amyloid.

In the present study, Aβ at the concentration of 20 µM decreased neuronal survival compared to the control-treated group (*P* < 0.001; Figure 1). In contrast, memantine (2.5 µM) and dizocilpine (5 µM) suppressed the neurotoxic effects of Aβ on the SH-SY5Y cells *P* < 0.001) (Figure 1). Moreover, chloroquine (10 and 20 µM, *P* < 0.001 and *P* < 0.05, respectively) and BD-1063 (1, 10 and 30 µM, *P* < 0.001) decreased the neurotoxic effects of Aβ on the SH-SY5Y cells (Figure 1 and Figure 2). However, chloroquine (40 µM) increased the neurotoxic effects of Aβ (*P* < 0.001; Figure 1).

The findings of this study showed that the NMDA antagonists such as memantine and dizocilpine inhibited the neurotoxic effects of Aβ. *In vitro* and *in vivo* experiments have shown that memantine reduces the neurotoxic effects of Aβ.^23^ This effect might be due to the behavioral effects of memantine.^4^ Moreover, Harkany et al^23^ have shown that dizocilpine diminished the Aβ-induced neurotoxicity. The over-activation of NMDA receptors may mediate the neurotoxic effects of Aβ, and the inhibition of NDMA receptors suppresses this neurotoxic effect. Thus, the NMDA receptor antagonists have potential roles in the AD amelioration.^24^ However, the cellular process of neuroprotection produced by the NMDA antagonists is relatively unknown.

Our study showed that the lower concentrations of chloroquine (10 and 20 μM) suppressed the neurotoxic effects of Aβ. In contrast, chloroquine (at a higher concentration: 40 μM) increased the neurotoxic effects of Aβ. According to previous studies, chloroquine has produced both neuroprotective or neurotoxic effects. The low concentration of chloroquine had no apparent effect on the lysosomes and produced neuroprotective effects in the different models.^25,26^ In contrast, chloroquine at the concentrations higher than 25 μM decreased primary telencephalic neuronal viability.^27^ Another *in vitro* study has also demonstrated the neurotoxic effects of chloroquine at concentrations higher than 25 μM).^28^ Accordingly, chloroquine may have detrimental or beneficial effects on neurons.

### Chloroquine effects on the memantine and dizocilpine neuroprotective effects

Cell viability in the SH-SY5Y cells treated with memantine, dizocilpine and chloroquine were different (F (12,39)= 47.764, *P* < 0.001) (Figure 1). Accordingly, cell viability in the cells treated with memantine + Aβ + chloroquine (10, 20, and 40 μM) was significantly lower than the memantine + Aβ-treated group (*P* < 0.001) (Figure 1). Moreover, cell viability in the memantine + Aβ + chloroquine (20 μM) group was higher compared to the Aβ + chloroquine (20 μM) group (*P* = 0.025) (Figure 1). Our study also showed that cell viability in the dizocilpine + Aβ + chloroquine (10, 20, and 40 μM) was significantly lower than the dizocilpine + Aβ-treated group (*P* < 0.001; Figure 1). Moreover, cells treated with Aβ + chloroquine (40 μM) showed lower cell viability compared to the dizocilpine + Aβ + chloroquine (40 μM)-treated group (*P* = 0.004; Figure 1). The present study showed that cell viability in the memantine + Aβ + chloroquine (20 μM) group was higher than the dizocilpine + Aβ + chloroquine (20 μM) group (*P* = 0.006; Figure 1).

The inhibition of lysosomal activity may be related to the neurotoxic effects of chloroquine. Lysosome dysfunction leads to Aβ accumulation and enhances neurotoxicity.^29^ Simultaneously, Aβ causes lysosomal dysfunction in the SH-SY5Y cells.^30^ Moreover, the neurotoxic effects of Aβ are similar to lysosomotropic agents.^30^ On the contrary, the pharmacologic induction of autophagy produces neuroprotective effects against proteinopathies.^31^ Chloroquine is a weak base that disrupts the lysosome activity in neurons by the inhibition of autophagosomes inhibition to the lysosome.^32^ Therefore, the chloroquine inhibitory effects on lysosome may block the Aβ degradation and increase the Aβ-induced neurotoxicity. In this regard, *in vivo* studies have shown that lysosomal inhibition may lead to the reduction of Aβ degradation and neurodegeneration.^26,33^ Therefore, the chloroquine effects on lysosome especially at the higher concentrations overcomes the neuroprotective effects of this agent and potentiates the neurotoxic effects of Aβ.

There are some controversies regarding the memantine effects on the autophagy. In a study conducted by Yoon et al, the authors reported that a high concentration of memantine activated the autophagy and apoptosis in the glioma cell lines.^34^ In contrast, memantine has decreased autophagy in a cellular model of the AD.^15^ The difference may be due to the dose of memantine and the cells used in these two experiments. However, there are limited data about the memantine effects on the lysosomal activity. According to our study, lysosome and autophagy inhibition decreased the neuroprotective effects of memantine and dizocilpine. Therefore, autophagy activation and the normal lysosomal function may be protective against the Aβ-induced neurotoxicity. In this regard, the autophagy induction has potentiated neuroprotection against Aβ-induced neurotoxicity in the SH-SY5Y cells.^35^ Accordingly, the NMDA receptor activation may cause lysosomal defect and magnify the Aβ neurotoxic effects. It is possible to assume that the NMDA antagonists may modulate the lysosome activity and autophagy in the process of neurodegeneration.

### BD-1063 interference with the memantine and dizocilpine neuroprotective effects

The present study showed that cell viability in the SH-SY5Y cells treated with memantine, dizocilpine, and BD-1063 was different (F (12, 39)=14.150, *P* < 0.001; Figure 2). In this regard, cell viability in the memantine + Aβ group was higher than the memantine + Aβ + BD-1063 (10 and 30 μM) groups (*P* = 0.003 and *P* = 0.002, respectively; Figure 2). Furthermore, cell viability in the Aβ + BD-1063 (30 μM) group was lower than the memantine + Aβ + BD-1063 (30 μM) group (*P* = 0.020; Figure 2). Our study also showed that cell viability in the dizocilpine + Aβ group was higher compared to the dizocilpine + Aβ + BD-1063 (1, 10, and 30 μM) (*P* = 0.010, *P* = 0.032, and *P* < 0.001, respectively) (Figure 2). Furthermore, cell viability in the Aβ + BD-1063 (10 μM) group was significantly lower than the dizocilpine + Aβ + BD-1063 (10 μM) group (*P* = 0.021; Figure 2). Cell viability in the memantine + Aβ + BD-1063 (30 μM) group was higher than the dizocilpine + Aβ + BD-1063 (30 μM) group (*P* = 0.035).

This study showed that a sigma receptor antagonist suppressed the Aβ-induced neurotoxicity. There are some inconsistencies in the literature about the neuroprotective or neurotoxic effects of sigma receptors. Some reports have shown that the sigma receptor agonists have produced neuroprotective effects in the neurodegenerative disorder models.^18^ On the other hand, BD1063, as a selective sigma receptor antagonist, protected against methamphetamine-induced neurotoxicity in the rat.^36^ In vitro and in vivo studies have also shown that AC927, another sigma receptor antagonist, prevented the methamphetamine-induced neurotoxicity.^37^ Therefore, the inconsistencies may be related to the models and the neurotoxic agent have been used in these experiments.

Our study showed that a sigma receptor antagonist decreased the neuroprotective effects of two NMDA antagonists against Aβ-induced neurotoxicity. Thus, the activation of sigma receptors may potentiate the neuroprotective effects of memantine and dizocilpine. Other studies have shown that the activation of sigma receptors prevented glutamate-mediated neurotoxicity.^38^ In contrast to our results, some studies have shown that the sigma receptor agonists have potentiated the glutamate activity by increasing the expression of the NMDA receptors subunits and the plasma membrane level of NDMA receptors.^22^ These inconsistencies may be related to the different interactions between NMDA receptors and sigma receptors in the normal and degenerative conditions.

Our study had some limitations. We did not measure the lysosomal function and NMDA receptor trafficking in the treated cells. Moreover, some agents may have non-specific effects. Thus, future studies may help to discover the cellular interaction between the NMDA antagonists and the lysosome and sigma receptor functions in the process of neurodegeneration.

## Conclusion

The lysosomal function and sigma receptors may contribute to the neuroprotective mechanism of memantine and other NMDA receptor antagonists. Moreover, the restoration of lysosomal function and the modulation of sigma receptors may be potential strategies in the treatment of AD. Future studies may help to discover the exact contribution of lysosome and sigma receptor functions to the neuroprotective effects of the NMDA receptors.

## Ethical Issues

Not applicable.

## Conflict of Interest

Non-declared.

## Acknowledgments

We gratefully thank the Vice-chancellor for research affairs of the Shiraz University of Medical Science for the financial support. It is important to note that sponsor had no role in the design of the study and collection, analysis, and interpretation of data and in writing the manuscript.
